# A Cost-Affordable Methodology of 3D Printing of Bone Fractures Using DICOM Files in Traumatology

**DOI:** 10.1007/s10916-024-02084-w

**Published:** 2024-07-08

**Authors:** Kristián Chrz, Jan Bruthans, Jan Ptáčník, Čestmír Štuka

**Affiliations:** 1 1st Surgical Department, General Teaching Hospital, Prague, Czech Republic; 2 Department of Biomedical Technology, Faculty of Biomedical Engineering, Czech Technical University in Prague, Prague, Czech Republic; 3 Department of Anesthesiology and Intensive Care, General Teaching Hospital, Prague, Czech Republic; 4 Institute of Biophysics and Informatics, 1st Medical Faculty, Charles University, Prague, Czech Republic

**Keywords:** 3D printing, Surgery, Medical education, Image-guided surgery, Patient-specific models, Preoperative planning

## Abstract

Three-dimensional (3D) printing has gained popularity across various domains but remains less integrated into medical surgery due to its complexity. Existing literature primarily discusses specific applications, with limited detailed guidance on the entire process. The methodological details of converting Computed Tomography (CT) images into 3D models are often found in amateur 3D printing forums rather than scientific literature. To address this gap, we present a comprehensive methodology for converting CT images of bone fractures into 3D-printed models. This involves transferring files in Digital Imaging and Communications in Medicine (DICOM) format to stereolithography format, processing the 3D model, and preparing it for printing. Our methodology outlines step-by-step guidelines, time estimates, and software recommendations, prioritizing free open-source tools. We also share our practical experience and outcomes, including the successful creation of 72 models for surgical planning, patient education, and teaching. Although there are challenges associated with utilizing 3D printing in surgery, such as the requirement for specialized expertise and equipment, the advantages in surgical planning, patient education, and improved outcomes are evident. Further studies are warranted to refine and standardize these methodologies for broader adoption in medical practice.

## Introduction

While three-dimensional (3D) printing has become widespread in both professional and amateur domains, its integration into the field of medicine, particularly in surgery, remains largely confined to individual projects and a limited number of case studies. The main reason is that the 3D printing method is still not as “user-friendly” as one might expect and certainly not on the same level as printing a document on an office paper printer. Specifically, the process of transferring Computer Tomography (CT) images (DICOM files - Digital Imaging and Communications in Medicine) to a 3D file (.stl file - STereoLithography), which must be further processed before final printing, is quite complicated. Unfortunately, this procedure necessitates the use of multiple distinct programs, adding a layer of complexity. Moreover, a certain level of knowledge of 3D printing and 3D modeling is required.

3D printing is widely used in the field of medicine, particularly in traumatology. However, most available publications focus on specific cases or the use of already-made 3D models. Consequently, the literature lacks comprehensive, step-by-step instructions on converting DICOM images from CT scans to .stl files, processing them, and preparing them for final printing. This workflow is still largely explained by amateur enthusiasts and documented on forums dedicated to hobbyist 3D printing.

The study aims to create a straightforward, clear, easy-to-understand, and repeatable guide for converting DICOM files to .stl format, modifying them, preparing them for 3D printing, and executing the print, all while emphasizing the use of available software and keeping costs minimal. This tutorial is intended for clinicians who wish to venture into the field of 3D printing.

We present the individual steps as guidelines, offering estimates of time requirements, approximate costs, technical background, and necessary software. We prioritize free-use open-source programs to ensure that physicians adopting this methodology face no financial constraints, being limited only by their willingness and interest in embracing 3D printing. A section of tips-and-tricks is added to help readership starting with 3D printing. We also show the use of our methodology in our daily practice, including details such as the number of patients for whom we have employed this approach, outcomes, and other pertinent information.

The primary use of the resulting 3D printed models is for preoperative planning of complex intra-articular fractures. Additionally, these models are used to explain the specifics of the fracture to the patient, plan surgical incisions, and demonstrate the use of the chosen implant. Moreover, the models serve as an excellent teaching aid for both medical and rehabilitation students.

## History

The concept of 3D printing initially captivated the imagination of writers and fantasists. In 1964, A. C. Clarke foresaw a future where a machine known as a ‘replicator’ would effortlessly create objects, much like printing books. Hideo Kodama Nagoji was one of the first to develop a rapid prototyping technique using a single laser beam. In 1980 and 1981, he published papers on his experiments to develop methods for the automatic fabrication of three-dimensional models using UV light and light-sensitive resin. His method involved a mask to control UV source exposure, outlining techniques for solidifying thin successive layers of photopolymer, laying the groundwork for what he would later term stereolithography (SLA) [1, 2].

In a parallel development, Charles Hull introduced stereolithography in 1984. Two years later, he secured a patent for this technique, in which he described a process in which liquid polymers were solidified under UV light to form cross-sections of a 3D model. This method used digital data and a computer-controlled beam of light to create individual layers, one on top of the other. Hull subsequently founded 3D Systems Inc., which eventually began manufacturing and selling stereolithography machines. The world’s first commercial SLA printer was produced by 3D Systems in 1988 [1, 3].

In 2004, Dr. Adrian Bowyer founded RepRap, an open-source initiative promoting the production of low-cost, self-replicating 3D printers. This led to a greater proliferation of these 3D printers and a surge in their popularity among hobbyists. In 2008, the first such self-replicating 3D printer was produced. More manufacturers entered the market and the price of these printers began to drop radically when the patents protecting FDM (Fused Depositing Modeling) technology expired in 2009.

Since 2009, the first providers of 3D printing as a service have also emerged. FDM printers continued to gain more market share after 2010, not only among hobbyists. They are gradually becoming part of the product development process.

In early 2020, the Czech designer and producer of 3D printers Prusa Research gained recognition among healthcare professionals amid the COVID-19 pandemic by providing hospitals with 3D-printed shields and other protective gear at no cost. Around that time, we also obtained our first Prusa MK3S + printer and developed our own customized protective mask at our workplace. Our mask, constructed from polylactic acid (PLA) filament, featured a modular design tailored to the contours of the face and included adapters for standard anesthesia machine filters.

## Methodology

### Requirements for the CT Image

To print a 3D model of the fracture with attention to detail, access to CT images in detailed resolution is needed. A large number of departments due to lower data storage costs save a layer of the image every 5 mm, while the rest of the data is lost. Therefore, for our methodology, the acquisition of CT images at their original resolution is required, typically necessitating collaboration with local radiologists. According to our experience, images with a minimum resolution of 0.75 mm per layer are convenient for the final 3D print.

### Accessing and Transferring DICOM Images to a 3D File (.stl)

In the initial CT image processing step, known as image segmentation, window leveling is used to select bone tissue based on the Hounsfield unit (HU) scale. This is a common procedure for any radiologist operating a modern CT scanner. The HU level for bone typically ranges from approximately 1000 to 3000 [4].

- If a 3D reconstruction of the bone is accessible through the PACS viewer, it is apparent that the radiologists have already conducted this procedure, and they can furnish the exported .stl 3D model for subsequent steps. Collaborating with them in this regard can significantly save time in the long run.
- If there is no existing 3D reconstruction, the radiology professional software for medical image acquisition, processing, and storage can be used, as it allows easy and thorough processing including 3D reconstruction. In our hospital Philips IntelliSpace Portal (Philips Healthcare, Andover, MA, USA) is used, reconstruction in the bone window with trimming of excess parts is very intuitive for us. The final result can be exported as a single .stl file.
- If access to such software is unavailable, the procedure becomes more challenging. While many DICOM viewers in medicine allow the export of individual images, exporting an entire CT scan requires exporting 150 to 300 images individually. These images can then be uploaded to a freely available 3D slicer (https://www.slicer.org/), where reconstruction is performed, in our case, in the bone window with cropping of unnecessary parts. The final result is then exported to a .stl file.”

### Removing Invalid Data from the 3D File

The acquired 3D file needs to be cleared of invalid data, including unsealed areas and free fragments. The 3D model needs to be watertight, meaning it has closed surfaces. To achieve this, we use the freely available Meshmixer (Autodesk, Inc., San Francisco, CA, USA, https://meshmixer.com/) and utilize the ‘Import object/Analysis/Inspector/Auto-repair’ function, saving the repaired result in a .stl file.

### Connecting Free Parts in the 3D File

The 3D file from the previous step already contains bone reconstruction only. Therefore, successful printing is not technically feasible unless these individual bones, including their fragments in our case, are connected. This process typically involves the manual insertion of rollers and rods, which can be painstakingly tedious. Various 3D software options, including Meshmixer (Autodesk, Inc., San Francisco, CA, USA) or the open-source Blender (Blender Institute B.V., Amsterdam, Netherlands, https://www.blender.org/), as well as professional software like Rhinoceros3D (Robert McNeel & Associates, Seattle, WA, USA), 3D Studio MAX and AutoCAD (both Autodesk, Inc., San Francisco, CA, USA) etc., can be utilized for this task.”

### G-code Preparation for a Specific 3D Printer

The 3D printer-specific slicer (G-code producing software) should be used for this step. If no such software is available, we recommend using freely available PrusaSlicer (Prusa Research, Prague, Czech Republic, https://www.prusa3d.com/) or UltiMaker Cura (UlltiMaker, Zaltbommel, the Netherlands, https://ultimaker.com/).

The 3D file (.stl) is imported into the slicer software and the model is positioned on the print sheet. It is essential to verify that it fits; otherwise, the 3D model must be trimmed accordingly. If the trimming is performed by a physician, anatomical awareness can be assumed. However, if a technician handles the processing, consultation with a physician is necessary to ensure that the trimming does not compromise the anatomical integrity.

Since 3D printing is inherently spatial, it builds objects by placing one layer on top of another. However, if a part of the model does not rest directly on the printing sheet and instead starts in the air, it is referred to as an overhang in 3D printing terminology. In such cases, it is necessary to create supports for the model at these points. Supports can be generated automatically using slicing software and can also be manually adjusted if needed.

For practitioners new to 3D printing, we recommend using the Automatic Support Generation feature. Creating supports manually is suitable only for advanced users. If the supports are not properly created, the entire model may collapse during printing.

We recommend using “organic support” in PrusaSlicer, or “tree support” in UltiMaker Cura. These supports generally consume slightly less material, are easier to remove and the overall printing is usually slightly faster. Already in this phase, the 3D printing material must be chosen – we recommend using PLA Filament.

### 3D Printing

For reasons of price-performance ratio, relatively low printing costs, and adequate printing area, we chose a 3D printer of the FDM (Fused Deposition Modeling) type. We also considered using an SLA (Stereolithography) or SLS (Selective Laser Sintering) printer.

FDM is generally the cheapest and simplest method, but its models are less detailed and may require additional adjustments and cleaning. SLA provides highly detailed results and is ideal for printing complex models with fine details. However, this method requires more maintenance, and the resin can be more expensive than the filament used in FDM printing. SLS is the most advanced of the three methods and produces the highest quality and strongest models. However, the printing process is slower, and the material and operating costs are higher.

When starting an FDM printing, the print sheet should be entirely free of grease, and this is achieved by using technical alcohol or acetone. Depending on the speed of the printer and the size of the model, a print time varying between 15 and 50 h can be expected! After printing the model, supports are removed using pliers. Even when all countermeasures are thoroughly implemented on a complex model, approximately 20% of print failures occur, which will increase the overall production time of the model.

### Usage of the Printed 3D Model

Over time, we have devised various applications for the utilization of printed 3D models, including surgical planning, educational purposes for students, and conveying the nature of injuries to patients.

## FAQ, Tips, and Tricks

### 3D Printer

There are a large number of cheap and affordable FDM-type 3D printers on the market. Immediate investment in a high-tech professional 3D printer is not necessary; comparable results can be attained with affordable unbranded printers, though initial challenges may need to be overcome. Nevertheless, we recommend opting for a 3D printer that has available support, accessories, and spare parts as well as an active discussion forum. This ensures assistance with individual faults, errors, repairs, and settings. The Prusa printers (Prusa Research, Prague, Czech Republic), specifically the i3 MK3S + and MK4 models, have proven to be exceptionally successful in our context.

### 3D Filament

When using an FDM printer, the choice of printing material is crucial, as poor-quality material can lead to numerous printing issues. Also, the color that is added to the filament affects its physical properties such as the resulting strength, etc. We recommend using a quality-proven filament, in our case, we use Prusament, Aurapol, PM Filament, etc.

**PLA** (polylactic acid) is a new type of biodegradable material derived from starch extracted from renewable plant resources. Due to its good processability and biodegradability, PLA 3D printer filament has become one of the most commonly used 3D printing materials. However, rapid biodegradability is only possible in an industrial composter and this material cannot be recycled together with other plastic materials.

As an alternative option, **PETG** can be used. PETG filament, also known as Polyethylene Terephthalate Glycol, is a co-polyester recognized for its durability and ease of use. The G in PETG stands for glycol-modified, which makes the end product clear with glass-like visual properties.

The advantage is easy recycling with the possibility of repetition. However, preparing a printing sheet can be more intricate for beginners.

Another commonly employed material in the market is **ABS**, Acrylonitrile Butadiene Styrene. ABS is generally used for making inexpensive, sturdy mechanical parts (LEGO bricks, car parts, cellphone parts, etc.). The material is tenacious and temperature resistant - this makes it suitable for engineering applications. ABS printing requires specific conditions for successful results. It’s important to conduct the printing in a well-ventilated room due to **the release of fumes and substances that may pose potential health risks**. A big problem is the cracking of the model during cooling, which requires a higher stable temperature around the printed model.

### Model Selection

For beginners, it is advisable to avoid starting with complex models that require several hours of print time. We recommend beginning with small models and toys unrelated to medicine that can be printed within minutes. This approach helps to prevent the frustration and demotivation that can occur if complications and glitches arise during a lengthy print job. Investing 20 h or more in a print that ultimately fails can be particularly discouraging.

### Multi-Material Print

Certain printers offer the capability for multi-material printing, allowing for the use of multiple filaments of the same type but in different colors, as well as multiple materials. For example, supports can be printed using water-soluble material. While this may seem tempting, we consider this approach suitable for advanced users only due to its increased complexity, leading to a significantly higher error rate. Only after these initial setbacks have been mastered do we recommend moving forward with printing more complex models or experimenting with multi-material print.

### Local Database

We highly advise cataloging and storing all copies in a local database from the outset, along with devising a method for labeling individual models. As the number of 3D models increases, it becomes exceedingly challenging to track down the source CT scans for each model.

### Size of Printed Model

When printing a larger model, it is important to consider the size limitations of the specific 3D printer being used. For instance, the Prusa MK4 offers a print space of 250 × 210 × 210 mm. If a bone model exceeds these dimensions, there are a couple of options available. Firstly, the model can be divided into two parts using for example PrusaSlicer software, positioned side by side on the printing bed, and printed simultaneously. Subsequently, the parts can be joined together through post-processing by gluing. Alternatively, the model can be rotated diagonally instead of being printed horizontally. This approach can provide approximately 380 mm of printing height, allowing for larger models to be printed with transparency. Another option is to utilize a printer with a larger print area, such as the PrusaXL, which offers dimensions of 360 × 360 × 360 mm, or diagonally nearly 619 mm. However, it’s important to note that the cost of such a printer is approximately 2200 USD.

Most intra-articular fractures are less than 200 mm in length, however, for example, in the case of a complex fracture of the proximal and distal tibia, a length of more than 450 mm must be considered.

### Time Requirements

The required time may vary depending on the complexity of the project; therefore, the following overview should be considered approximate:

1. Primary model creation using 3D Slicer, Philips Intelispace, or other – 2 h.
2. Invalid data processing in Meshmixer – 10 min.
3. Connecting the parts and fragments of the model – 2 h.
4. G-Code creation (depends on the CPU speed) – 10 min.
5. Printing time (according to model complexity) – 15 to 50 h.
6. Finishing the 3D model and removal of supports – 10 min.

The entire process involves approximately 5 h of design time, which can be shortened if a 3D model already exists or if a radiologist is willing to prepare one. However, the primary time investment lies in the printing phase. The choice of the specific printer and its print nozzle size, layer, and support settings becomes crucial. In our case, we conducted a comparison between an older model Prusa printer (i3 MK3S+) and a newer model (MK4). With the printer set to default specifications (nozzle 0.4 mm, layer size 0.2 mm, vertical shell perimeter 2), we achieved the following models with corresponding printing times and filament consumption:

#### Ankle Joint Model

- i3 MK3S+, grid support: 28 h 01 min, 214 g / 72 m of filament.
- i3 MK3S+, organic support: 27 h 26 min, 191 g / 64 m of filament.
- MK4, grid support: 15 h 16 min, 213 g / 71 m of filament.
- MK4, organic support: 15 h 26 min, 195 g / 65 m of filament.

#### Base of the Skull Model

- i3 MK3S+, grid support: 59 h 38 min, 492 g / 165 m of filament.
- i3 MK3S+, organic support: 56 h 8 min, 368 g / 123 m of filament.
- MK4 grid support: 29 h 10 min, 454 g / 152 m of filament.
- MK4 organic support: 31 h 00 min, 389 g / 130 m of filament.

While the filament consumption does not differ significantly, the newer printer was able to reduce the required time by almost half. The selected supports did not have a substantial effect on the printing time.

Since this methodology is primarily intended for physicians, it is understood that their primary responsibility is the standard treatment of patients. Creating 3D models, especially in the initial stages, falls outside their usual scope of work and consumes valuable time. Therefore, the time required for this process cannot be overlooked. However, if the task of processing images into 3D models is delegated to a technician, this time constraint is significantly reduced.

### Cost-Effectiveness

While there are notable price differences among individual filaments, their selection, as mentioned above, is crucial. The cost for 1000 g of PLA filament typically falls within the range of 17–30 USD. For reference, an ankle joint model weighs about 200 g, and a skull base model weighs about 500 g.

## Results

In our hospital, we began utilizing 3D printing as an auxiliary method for treating complex intra-articular fractures in 2020. Over several years, we have refined and developed this methodology, identifying suitable free software to encompass the entire process. We have also perfected the 3D model preparation, enabling us to typically complete the entire process error-free on the first attempt. To date, we have successfully created 72 models, as listed in Table 1.

**Table 1:** **3**D models created in our department using the methodology

Type of model	Quantity
Shoulder joint model	5
Elbow joint model	7
Knee joint model	15
Tibia model	5
Ankle joint model	25
Heel bone model	10
Wrist model	5
**Total**	**72**

Throughout our usage, the resulting models have served various purposes:

- Enhancing the planning of surgical approaches, determining individual incisions, and aiding in the selection of appropriate implants for complex atypical fractures.
- Serving as teaching models in medical education for students.
- Acting as presentation models to explain surgical approaches, options, and the extent of fractures to patients. Unlike radiographs or CT scans, which only a small number of patients can interpret regarding the extent and severity of a fracture, a solid and tangible 3D model of the bone provides an easy visualization for most patients.

### Case Series

Each case is illustrated with a separate figure, each consisting of three parts: (a) radiograph, (b) 3D reconstruction, and (c) 3D print of the fracture.

#### Case 1

**Fig. 1:**
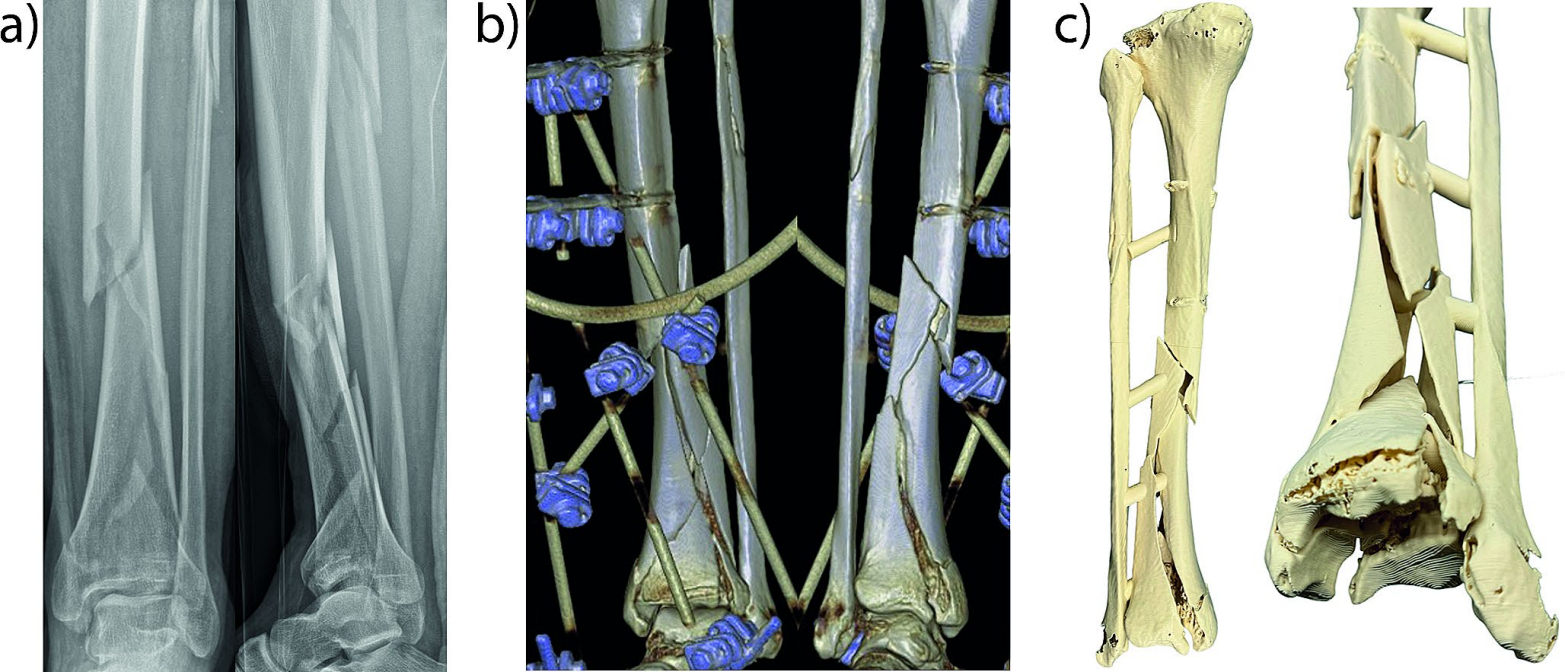
Radiograph, 3D reconstruction, and 3D print of an open fracture of the tibia (Case 1)

(Fig. 1) A 33-year-old delirious patient sustained an open fracture of the tibia, classified as Type II according to the Gustilo classification, after jumping from the 2nd floor. The fibula fracture was classified as 4F1A and the tibia fracture as 43C3.3 according to the AO classification system. The initial treatment involved the application of an external fixator. Additionally, a CT reconstruction of the fracture was conducted, and a 3D model of the fracture was created to facilitate better planning, particularly due to significant fragment dislocation around the tibial joint.

#### Case 2

**Fig. 2:**
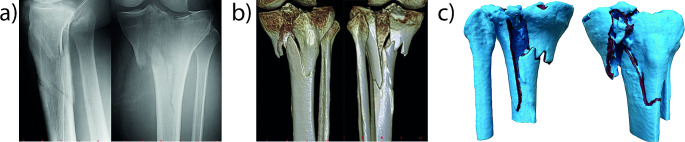
Radiograph, 3D reconstruction, and 3D print of an unusual proximal tibia fracture (Case 2)

(Fig. 2) A 60-year-old female patient exhibited an unusual proximal tibia fracture, which included involvement of the medial plateau, avulsion of the medial condyle, and an oblique fracture of the proximal metaphysis. The fracture classification most closely resembled AO classification 41C3.2 or Schatzker type IV. To enhance the precision of planning for the two approaches, a 3D printed model derived from CT reconstruction was utilized.

#### Case 3

**Fig. 3:**
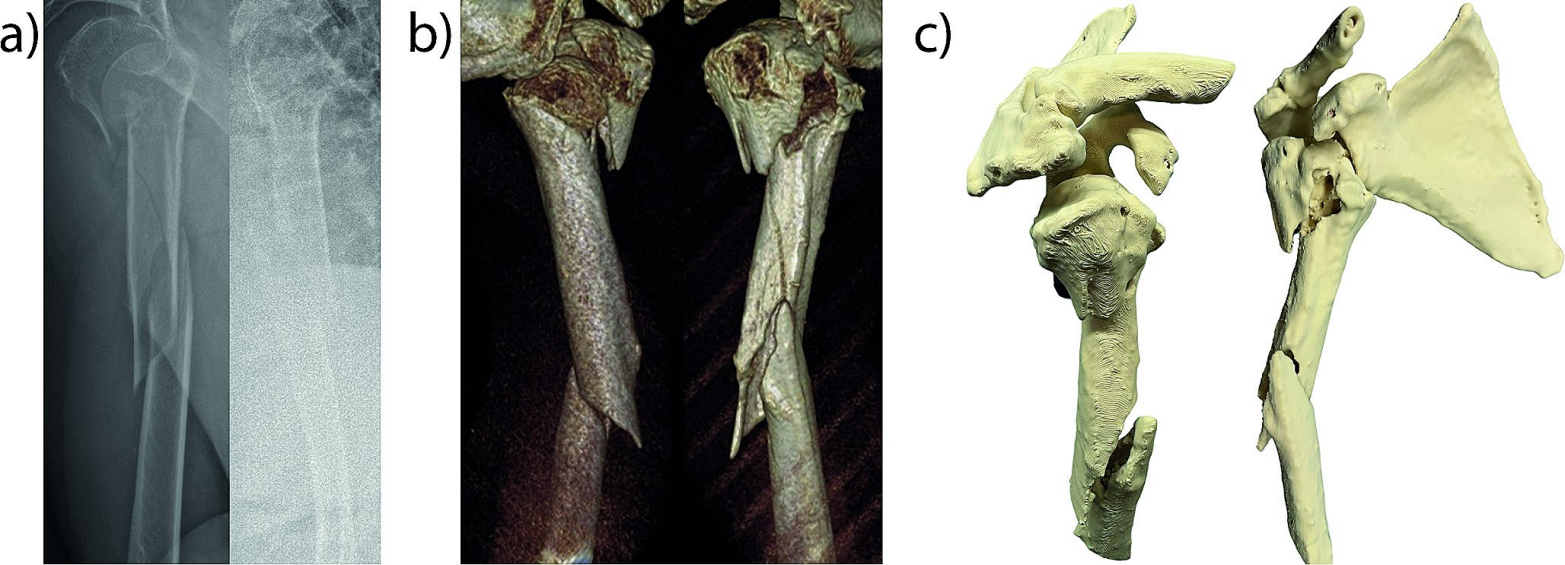
Radiograph, 3D reconstruction, and 3D print of a proximal and diaphyseal humerus fracture (Case 3)

(Fig. 3) A 78-year-old female patient, sustained a proximal humerus fracture (11C3.2) and a diaphyseal humerus fracture (12A1) according to the AO classification, following a fall at home. As part of the preoperative preparation, a CT reconstruction was conducted due to the complex nature of the fractures, followed by the printing of a model to aid in spatial visualization.

#### Case 4

**Fig. 4:**
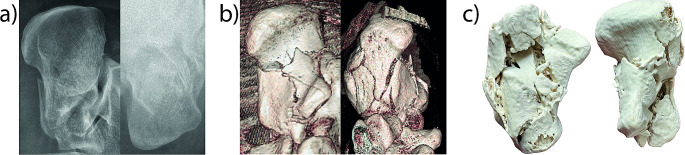
Radiograph, 3D reconstruction, and 3D print of a triple fracture of the calcaneus (Case 4)

(Fig. 4) A 53-year-old patient suffered a triple fracture of the calcaneus after falling 2 m from a ladder. The CT scan revealed a fracture with joint depression with 0 Bohler angle, classified as 3BC according to Sanders classification. Neither the radiographs nor the subsequent CT reconstruction sufficiently predicted the shape of the fragments. Only the 3D-printed model provided a substantial improvement in spatial representation.

## Discussion

The use of 3D printing in medicine, especially in surgery and traumatology, is well-documented and quickly advancing. However, many medical practitioners are still unfamiliar with the specialized software and 3D modeling techniques. We aim to bridge this gap by offering a comprehensive methodology and clear instructions for converting image documentation into final material models using a 3D printer.

Time is regarded as the primary limitation in the utilization of 3D printing in surgery [5]. The time frame for printing is noted to range from 10 h to 2 weeks [5–7]. A wide variance is influenced by factors such as the complexity and size of the printed model, as well as the speed of the printer itself. In our practice, we have managed to reduce printing times by half by employing a newer model printer from the same manufacturer, while keeping the price of the printer roughly the same. However, it should be noted that technical issues are not uncommon, particularly with such large models. The most frequent interruptions in printing occur due to power failures, shifting or jumping in the XY axis, and detachment from the printing sheet caused by contraction of printing material during cooling and by forces caused by the printing head. In such cases, printing times within a week are conceivable.

Another limitation of utilizing 3D printing is the cost, as the acquisition expenses for software and hardware typically range between 12,000 and 40,000 USD [8]. To mitigate these costs, we opt for open-source free software and select a printer with a modest purchase price yet satisfactory print quality. As a result, our acquisition expenses are significantly reduced, not exceeding 700 USD.

We agree that for the routine integration of 3D printing into the practices of surgeons and trauma specialists, the necessity to navigate through numerous steps involving collaboration with various disciplines and specialists may pose the most significant limitation [9]. Handling imaging and reconstruction software for DICOM files, alongside proficiency and familiarity with 3D printing software, demands specific IT expertise that most surgeons simply lack. In our experience, even within a large surgical department comprising approximately 50 surgeons, only a few individuals possess the capability to work with such software. Additionally, reluctance among senior colleagues to adopt these novel treatment methods could also be a contributing factor.

The literature describes better patient compliance in the postoperative course and during rehabilitation. Preoperative planning utilizing 3D fracture models has shown shorter operative times, reduced blood loss, and improved elbow function compared to conventional surgical planning based on two-dimensional image data [10–12]. In our experience, educating patients about upcoming surgeries is significantly easier using printed 3D fracture models than conventional imaging, which many patients have a limited understanding of. 3D printing is not limited to surgery applications, for example, it was used previously to evaluate the feasibility of one-lung isolation in a pediatric patient [13].

The teaching of anatomy traditionally relies on cadaveric dissections and anatomical atlases. However, in literature, anatomical structures are typically depicted from a planar view, making it challenging to grasp spatial awareness adequately. Cadaveric dissection provides a tangible learning experience, but cost constraints and limited body donations in certain countries result in high student-to-cadaver ratios [14]. The emergence of 3D printing offers a compelling solution by providing cost-effective, highly accurate, and tangible representations of anatomical structures, surpassing the limitations of traditional methods [15]. This technology extends beyond bones to encompass various body structures, enhancing the educational experience [16]. There are numerous models of physiological bones available for anatomy and teaching purposes, but only a few depict recent unhealed fractures, which can be easily produced using this method.

Limitations of our methodology include challenges related to access to DICOM source files. Additionally, communication and collaboration between radiologists and surgeons may be difficult, further complicating the process. Another obstacle is the acquisition of a 3D printer and the funding required for its operation, particularly concerning the purchase of filament. Since 3D printers are not yet classified as medical devices, it might be necessary to utilize grants to procure them, or they could be acquired by medical faculties as teaching aids. The ability to engage in 3D printing during working hours rather than personal time, such as nights and weekends, depends on clinic management policies. Ultimately, however, the primary and most significant limitation rests on the human factor. The methodology relies on early adopters - physicians who possess an interest, expertise, and education in computer technologies, data processing, graphical programs, and ultimately, 3D printing.

## Conclusions

This methodology for 3D printing bone fractures from DICOM files should be considered an initial suggestion only. We believe it has broad applicability, such as in preoperative planning, patient comprehension, and student education. By utilizing free, open-source software and providing detailed step-by-step guidelines, the 3D printing process becomes accessible to clinicians without extensive expertise in this field and even with financial constraints. However, each workplace may need to adjust the presented methodology according to its capabilities. Further studies are necessary to establish a universal methodology for the expanded use of 3D printers in surgery. While commercial solutions may not be as cost-effective, they could prove beneficial for the widespread adoption of these novel technologies.

## Data Availability

No datasets were generated or analysed during the current study.
